# Stromal-epithelial interaction induces *GALNT14* in prostate carcinoma cells

**DOI:** 10.3389/fonc.2023.1212585

**Published:** 2023-08-21

**Authors:** Elena D. Czyrnik, Marc Wiesehöfer, Jaroslaw T. Dankert, Sven Wach, Mathias Wagner, Martin Spahn, Marianna Kruithof de Julio, Gunther Wennemuth

**Affiliations:** ^1^ University Hospital Essen, Department of Anatomy, Essen, Germany; ^2^ Department of Urology and Pediatric Urology, University Hospital Erlangen, Erlangen, Germany; ^3^ University Hospital Saarland, Department of General and Special Pathology, Homburg, Germany; ^4^ Lindenhofspital Bern, Department of Urology, Bern, Switzerland; ^5^ University Hospital Essen, Department of Urology, Essen, Germany; ^6^ Department for BioMedical Research, Urology Research Laboratory, University of Bern, Bern, Switzerland; ^7^ Department of Urology, Inselspital, Bern University Hospital, Bern, Switzerland

**Keywords:** prostate tissue, prostate carcinoma, stromal-epithelial interaction, paracrine interaction, co-culture, GALNT14

## Abstract

**Introduction:**

Cell-cell communication is an important process in healthy tissue but also gains enhanced attention regarding pathological tissue. To date, the tumor microenvironment is gradually brought into focus when studying tumorigenesis. In the prostate gland, stromal and epithelial cells greatly interact to maintain homeostasis or tissue integrity. This study focuses on an indirect communication via soluble factors.

**Methods:**

To investigate the cell-cell interaction via soluble factors, the prostate carcinoma cell line LNCaP and the stromal primary cells p21 were co-cultured without direct contact and RNA was isolated at defined time points. Differences in gene expression were finally analyzed by RNA sequencing.

**Results:**

RNA sequencing revealed a time-depending differential expression profile. Selected factors were subsequently characterized at molecular level and analyzed in human prostate tissue of different developmental stages as well as pathology. GALNT14 was one of the highest induced co-culture-specific genes in LNCaP cells. Detection in healthy tissue and BPH revealed an age-dependent decrease in GALNT14 expression. Moreover, in prostate carcinoma, GALNT14 expression heavily varied independent of the Gleason score.

**Conclusion:**

Overall, this work provides a basis for further studies related to paracrine stromal-epithelial interaction in prostate carcinoma and highlights the importance of GALNT14.

## 1 Introduction

The prostate gland is mainly composed of epithelial and stromal cells working in a close meshwork. This stromal-epithelial interaction is a relevant process during prostate development as well as in normal tissue and eventually in pathological prostate tissue. During organogenesis, mesenchymal cells induce the development of epithelial buds due to a paracrine action (1). It is thought that this interaction is reactivated especially in benign prostatic hyperplasia (BPH) and might also play a role in prostate cancer (PCa) (2). For a long time, the focus in the development of PCa was set on the neoplastic glandular epithelial cells themselves and the role of the adjacent stroma was underestimated. It is now well known that also the stroma, mainly composed of fibroblasts and smooth muscle cells, greatly contributes to carcinogenesis and cancer progression (3–5). This so-called reactive stroma shares similarities to the stroma at sites of wound repair but consists of cancer-associated fibroblasts (CAFs) among others (6–8). During physiological tissue repair and regeneration, reversible epithelial-mesenchymal transition (EMT) occurs in addition to the expression of various chemokines, cytokines, and matrix-modulating factors. EMT involves loss of cell-cell connection as well as cell polarity due to detachment from the basal lamina, which allows cells to migrate. CAFs can alter the microenvironment likewise but to support tumor development instead of abolishing malignant cells (9). Several studies highlight the relevance of stromal-epithelial interaction regarding a malignant degeneration of prostate cells (10, 11). It could be shown that the gene expression profile of stromal cells varies significantly depending on the presence of a tumor and on their zonal origin in the prostate (11–13).

Research is necessary to understand the molecular changes causing a mislead communication between epithelial and stromal cells and how this works in favor of the tumor. Since PCa is the second most common cancer in men worldwide according to Globocan 2020, research in tumor development, tumor progression and tumor heterogeneity is very important (14). This study focuses on the indirect paracrine interaction between prostate carcinoma cells and stromal cells to reveal new relevant pathways and target genes. The general gene expression pattern of the prostate carcinoma cell line LNCaP and the primary stromal cell line p21 after interaction was analyzed as well as the gene expression at different time points (day 1, day 3, day 7) and over time (comparison of day 1 to day 7). *GALNT14* could be revealed as one of the highest induced genes in LNCaP cells after paracrine interaction with p21 cells over time and its expression was further investigated in fetal, in healthy adult prostate tissue, in BPH, rhabdomyosarcoma of the prostate and finally in PCa with different Gleason scores. *GALNT14* encodes the eponymous N-acetylgalactosaminyltransferase and is one of 20 isoforms known to date (15, 16). The main function of the GALNT family is to initiate and regulate mucin-type O-glycosylation, which involves post-translational attachment of N-acetylgalactosamine (GalNAc) to the amino acids threonine and serine of proteins in the Golgi apparatus. GalNAc further serves as an attachment site for other enzymes to extend and branch the glycan chain (17, 18). O-glycosylation occurs commonly in mucins as they exhibit large repetitive serine and threonine domains (18). Overall, mucins act as a protective barrier not only for epithelial cells against inflammation or cellular stress, but also for tumor cells (19). This study provides new insights in the expression of GALNT14 in prostate carcinoma cells.

## 2 Results

### 2.1 Co-cultivation of stromal and epithelial prostate cells

**Figure 1:**
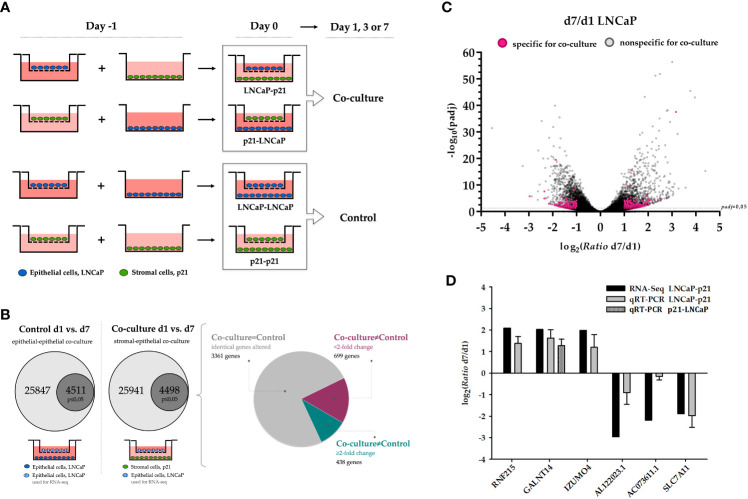
Gene expression changes in LNCaP cells via soluble factors after prolonged co-cultivation with p21 cells. **(A)** Cell culture inserts (Millicell, Merck) with a PET membrane and pore size of 1 μm were used to study the interaction between stromal (green) and epithelial (blue) cells via soluble factors. On day -1, cells were seeded either in the well of a 6-well plate or in the cell culture insert. On the following day (day 0), the co-culture system was formed by transferring the insert into the well and kept for one, three and seven day(s). The same cell type was co-cultivated as control in each case and RNA was isolated corresponding to the stromal-epithelial co-culture. **(B)** Schematic illustration of defining deregulated co-culture-specific genes in LNCaP cells. The proportion of significantly deregulated genes for control and co-culture is highlighted (p ≤ 0.05, dark grey). The pie chart additionally depicts the distribution of identical non co-culture-specific genes (gray, 3361 genes) as well as genes with less than a two-fold expression change (magenta, 699 genes) and at least two-fold induction or repression (cyan, 438 genes). **(C)** Relative expression change (ratio) as binary logarithm and the corresponding significance (adjusted p-value/padj) as negative decadic logarithm of all 25941 deregulated genes in LNCaP cells after co-cultivation for the comparison day 1 and day 7. The dashed line marks the limit of biological significance of p ≤ 0.05 (-log10>1.3). All 438 induced and repressed genes that were present exclusively as a result of co-cultivation and at least two-fold altered (from log_2_>1 and log_2_<-1, respectively) are highlighted (magenta). **(D)** Validation of RNA sequencing data by qRT-PCR of three independent co-culture experiments. Additionally, induction of *GALNT14* is also confirmed in reciprocal co-culture. Binary logarithm with standard deviation is indicated. d1, day 1; d7, day 7; padj, adjusted *p*-value; qRT-PCR, quantitative real-time PCR.

To investigate the stromal-epithelial interaction via soluble factors, the prostate carcinoma cells LNCaP were seeded into the well of a cell culture plate and primary stromal cells p21 into a corresponding hanging insert. Co-cultures with the same cell line were used as controls (**Figure 1A**). After one, three and seven day(s), RNA was isolated and quality controlled (**Figure S1A**) for subsequent gene expression profiling by 3’ RNA sequencing (n=4).

#### 2.1.1 Global gene expression changes induced by co-cultivation (all controls vs. all co-cultures)

First, the results from the controls of all three time points were summarized for the respective cell line and compared with the totality of all corresponding co-cultures. While the comparison of control and co-culture does not yield any significantly altered genes in LNCaP cells, there are a total of five induced genes in stromal p21 cells (p ≤ 0.05) (**Table 1**).

**Table 1:** List of deregulated genes in p21 cells after co-cultivation with LNCaP cells independent on the duration.

Ensembl-No.	Gene		Ratio^1^	*p*-value
ENSG00000247095	MIR210 Host Gene	*MIR210HG*	2.91	1.22·10^-11^
ENSG00000186352	Ankyrin Repeat Domain 37	*ANKRD37*	2.47	3.47·10^-10^
ENSG00000104419	N-Myc Downstream Regulated 1	*NDRG1*	2.22	9.96·10^-8^
ENSG00000116285	ERBB Receptor Feedback Inhibitor 1	*ERRFI1*	2.21	8.85·10^-9^
ENSG00000240583	Aquaporin 1	*AQP1*	2.06	2.11·10^-5^

^1^Relative expression change (co-culture vs. control).

#### 2.1.2 Differences in gene expression induced by co-cultivation at defined time points (control on day 1/3/7 vs. co-culture on day 1/3/7)

Next, gene expression changes at specific time points, i.e. day 1, 3 or 7, were considered separately. For this purpose, deregulated genes of the control on one day were compared with those of the corresponding co-culture. For LNCaP cells again, no significant gene expression alteration could be detected. For p21 cells, significant differences in expression are only apparent after three days (induction of nine genes and repression of two genes, p ≤ 0.05). After seven days, the number increases notably, especially for induced genes. Of a total of 98 deregulated genes (p ≤ 0.05), 86 are induced and 12 repressed. Gene ontology analysis was performed by using the bioinformatic databases DAVID and STRING. Eight of the 98 deregulated genes could thus be assigned to the “HIF-1 signal transduction pathway” (KEGG, hsa04066, EASE score =0.05) (**Table 2**).

**Table 2:** List of deregulated genes in p21 cells after co-cultivation with LNCaP cells for seven days attributed to the KEGG pathway “HIF-1 signal transduction pathway” (hsa04066).

Ensembl-No.	Gene		Ratio^1^	*p*-value
ENSG00000111674	Enolase 2	*ENO2*	4.03	1.83·10^-10^
ENSG00000109107	Aldolase/Fructose-Bisphosphat C	*ALDOC*	3.02	7.71·10^-6^
ENSG00000112715	Vascular Endothelial Growth Factor A	*VEGFA*	2.95	3.96·10^-14^
ENSG00000117394	Solute Carrier Family 2 Member 1	*SLC2A1/GLUT1*	2.52	3.67·10^-15^
ENSG00000129521	Egl-9 Family Hypoxia Inducible Factor 3	*EGLN3*	2.19	8.15·10^-3^
ENSG00000134333	Lactate Dehydrogenase A	*LDHA*	2.13	5.78·10^-5^
ENSG00000152256	Pyruvate Dehydrogenase Kinase 1	*PDK1*	2.00	2.77·10^-3^
ENSG00000072274	Transferrin Receptor	*TRFC*	0.48	9.33·10^-7^

^1^Relative expression change (co-culture day 7 vs. control day 7).

Furthermore, all deregulated genes from day 3 were also differentially expressed on day 7, except of CYP1B1 (**Figure S1B**).

#### 2.1.3 Longitudinal changes in gene expression induced by co-cultivation (control/co-culture on day 1 vs. control/co-culture on day 7)

Finally, this analysis aimed to reveal genes differentially expressed during prolonged co-cultivation times. Also, genes specifically induced or repressed due to co-cultivation rather than because of the duration of cultivation itself were revealed. For this purpose, we identified genes significantly deregulated during prolonged incubation periods (control day 7 vs. day 1) and excluded these genes from the analysis of genes significantly deregulated during prolonged co-cultivation (co-cultivation day 7 vs. day 1). Thus, we were able to define those genes that significantly changed in a longitudinal fashion upon co-cultivation. Below, this comparison is exemplarily explained in more detail for LNCaP cells. Results for p21 cells are shown in the supplement (**Figures S1C-E**; **Tables S1**, **S2**).

Comparing the controls on day 1 to the controls on day 7, 25847 genes match the reference list with a total of 60199 different genes (Ensembl release 98, September 2019). The comparison of the co-cultures on day 1 to day 7 results in 25941 matched genes. Taking a *p*-value of ≤0.05 into account, the number of differentially expressed genes decreases to 4511 and 4498, respectively. Last, the comparison of those two lists of genes reveals 1137 significantly altered genes due to co-cultivation, with 438 genes showing at least a two-fold induction or repression (**Figures 1B, C**).

**Table 3** summarizes the ten highest induced and repressed co-culture-specific genes (p ≤ 0.05), from which three each were validated by quantitative real-time PCR (qRT-PCR) in three independent co-culture experiments (**Figure 1D**). Candidate genes were chosen based on the strength of the expression change as well as scientific novelty regarding prostate tissue. For all six genes, induction or repression after seven days of co-cultivation could be confirmed corresponding to the RNA sequencing data (comparison of day 7 to day 1). This also approves the reliability of the sequencing data and the reproducibility of the co-culture experiment. *GALNT14* as highest induced gene among others was chosen as candidate gene for further analysis in prostate tissue. Moreover, it should be verified that the release of cellular soluble factors does not follow any orientation (apical or basal of the cell). Therefore, co-cultivation was also performed in reverse, i.e. LNCaP cells were seeded into the well and p21 cells within the hanging insert. Subsequent gene expression analysis by qRT-PCR again reveals the induction of GALNT14 expression after seven days of co-culture (n=3; **Figure 1D**).

**Table 3:** The 20 highest induced and repressed genes in LNCaP cells after co-culture with p21 cells depending on the duration (comparison of gene expression on day 7 to day 1).

Ensembl-No.	Gene		Ratio^1^	*p*-value
ENSG00000265972	Thioredoxin Interacting Protein	*TXNIP*	8.95	2.96·10^-38^
ENSG00000206885	Small Nucleolar RNA, H/ACA box 75	*SNORA75*	4.64	7.19·10^-4^
ENSG00000099999	**Ring Finger Protein 215**	***RNF215***	4.27	4.48·10^-5^
ENSG00000158089	**Polypeptide GalNac-Transferase 14**	***GALNT14***	4.08	1.80·10^-3^
ENSG00000029534	Ankyrin 1	*ANK1*	3.97	5.60·10^-5^
ENSG00000099840	**IZUMO Family Member 4**	***IZUMO4***	3.96	1.83·10^-5^
ENSG00000124587	Peroxisomal biogenesis factor 6	*PEX6*	3.80	6.65·10^-7^
ENSG00000161405	IKAROS family Zinc Finger 3	*IKZF3*	3.79	3.79·10^-3^
ENSG00000106665	CAP-Gly domain linker protein 2	*CLIP2*	3.73	0.00414
ENSG00000233836	Zinc Finger Protein 726 Pseudogene 1	*AC139769.1*	3.70	0.00228
ENSG00000278396	**lncRNA, Sense Intronic UNC79**	***AL122023.1***	0.13	1.70·10^-6^
ENSG00000222724	RNA, U2 small nuclear 63 Pseudogene	*RNU2-63P*	0.20	1.19·10^-5^
ENSG00000279207	new transcript	*AC015813.6*	0.20	2.40·10^-8^
ENSG00000257605	**MYG1 antisense RNA 1**	***AC073611.1***	0.22	1.05·10^-3^
ENSG00000232065	lincRNA 1063	*LINC01063*	0.26	2.73·10^-3^
ENSG00000228701	TNKS2 antisense RNA 1	*TNKS2-AS1*	0.27	4.67·10^-3^
ENSG00000258038	lincRNA 2327	*LINC02327*	0.27	4.79·10^-3^
ENSG00000196844	Prostate And Testis Expressed 2	*PATE2*	0.27	4.67·10^-3^
ENSG00000151012	**Solute carrier family 7 member 11**	***SLC7A11(xCT)***	0.27	3.64·10^-20^
ENSG00000136122	BORA aurora kinase A activator	*BORA*	0.28	2.00·10^-3^

^1^Relative expression change (day 1 vs. day 7 of co-culture). Highlighted genes were validated by qRT-PCR (bold face).

### 2.2 GALNT14 expression in non-malignant prostate tissue

**Figure 2:**
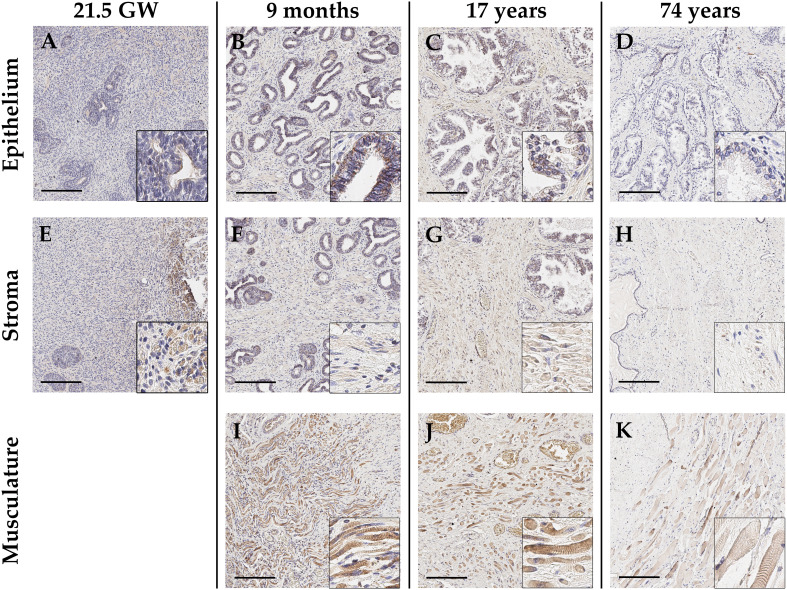
Representative immunohistochemical staining with anti-GALNT14 antibody of healthy prostate tissue differing in age, ranging from fetal over juvenile to adult (arranged column-wise). **(A-D)** Glandular epithelium is generally weakly positive for GALNT14. Only in pre-puberty prostate tissue **(B)** a distinct staining appears which gradually diminished with rising age. **(E-H)** The stroma only shows very weakly GALNT14-positive cells also exhibiting an age-related decrease. **(I-K)** Present skeletal muscle cells show a steadily strong GALNT14 expression. GW, gestation week; Scale bar, 200 µm.

In the context of prostate tissue, GALNT14 is poorly described and thus investigated in this study regarding its localization and distribution in non-malignant prostate tissue by immunohistochemical staining (**Figure 2**). Depending on the fetal development stage, the maturing glands are less branched and glandular tubes are not yet canalized and appear rather solid without the typical lumen. However, to ensure an unambiguous assignment and thus be able to make reliable statements about the GALNT14 expression, the epithelial marker cytokeratin 7 was detected first in the samples. In addition, the type 3 intermediate filament desmin was used to visualize muscle cells in the stroma (**Figure S2**). Next, GALNT14 expression was analyzed in fetal prostate tissue. According to eight samples from various gestation weeks (12; 14.5; 14.6; 17.1; 18; 18.2; 2x 21.5), GALNT14 is only weakly present in the epithelium and stroma (**Figures 2A, E, I**). The intensity and distribution of GALNT14 is equally reflected for all fetal prostate samples.

To examine an age-dependent expression, a broad range of prostate tissue from donors of different age were chosen. Regarding the glandular epithelium, GALNT14 expression tends to decrease with increasing age (**Figures 2A-D**). The prostatic epithelium of boys under one year of age (newborn, one month, nine months) shows a distinct staining, whereas it already declines in prostatic epithelium of the 17-year-old. Prostate samples from elderly men (56, 62, 63 and 74 years) finally show just a weak GALNT14 expression in the glandular epithelium of the prostate. Correspondingly, a decreasing expression in certain cells of the stroma could also be detected (**Figures 2E-H**). If skeletal muscle is present in the marginal areas of the tissue sections, a clear GALNT14 expression can be detected (**Figures 2I-K**). The same is true for perikarya of neurons, which appear strongly GALNT14-positive (**Figure S3E**). Furthermore, GALNT14 is detected in the endothelium of small vessels, although the staining here is variable (**Figures S3A-D**). In general, the staining of skeletal muscle, perikarya and vessels was constant, independent of age.

### 2.3 GALNT14 expression in benign prostatic hyperplasia (BPH) and rhabdomyosarcoma of the prostate

**Figure 3:**
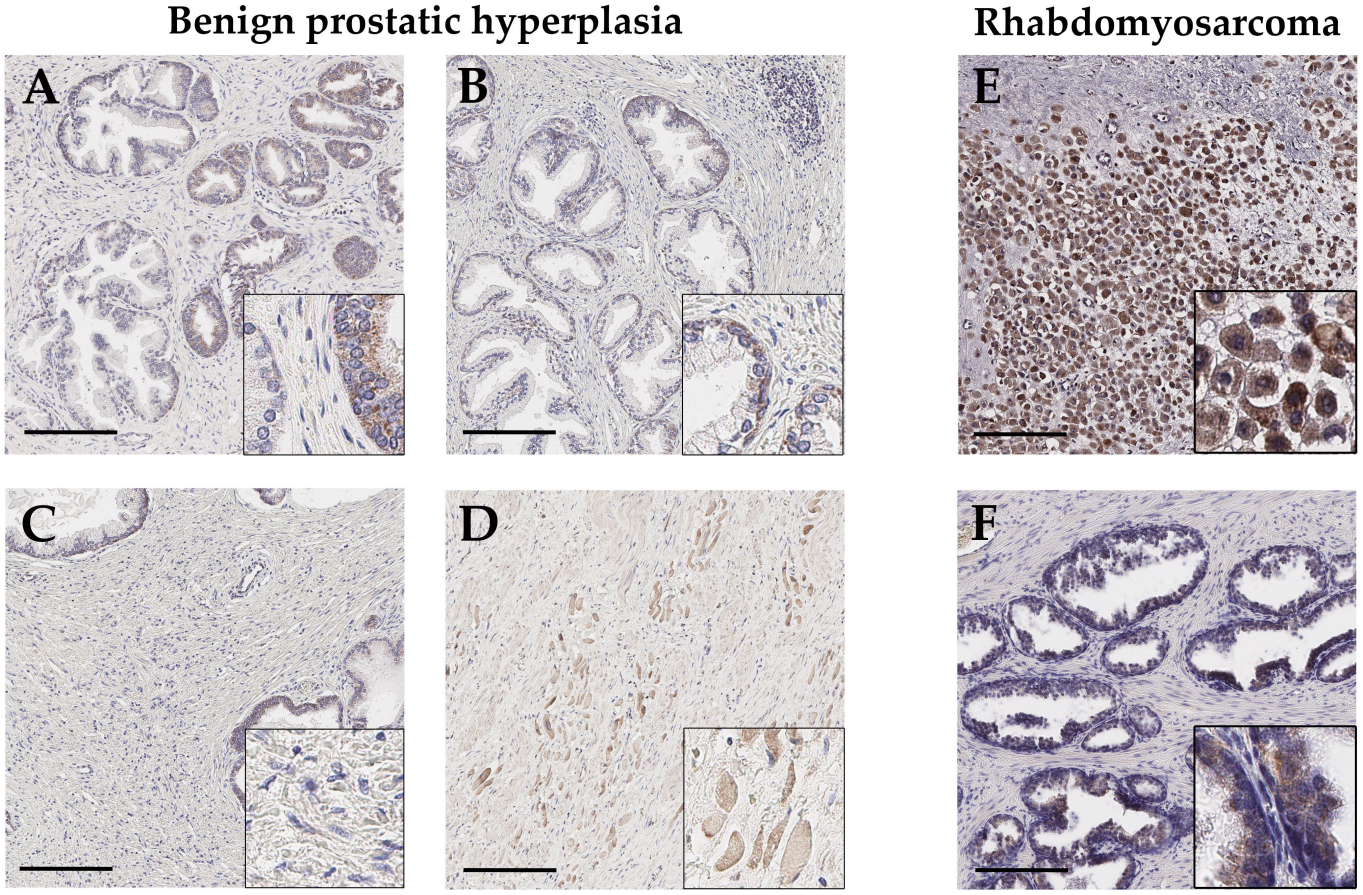
Representative immunohistochemical staining with anti-GALNT14 antibody of benign prostatic hyperplasia (BPH) and rhabdomyosarcoma. **(A, B)** Glandular epithelium shows weakly GALNT14 staining which is basally located or near the nucleus. **(C, D)** The stroma displays no staining for GALNT14, but adjacent skeletal muscle cells exhibit a distinct GALNT14 expression. **(E, F)** In rhabdomyosarcoma, tumor cells are strongly positive for GALNT14 while adjacent normal tissue shows the typically weak staining of glandular epithelium. Scale bar, 200 µm.

In BPH tissue (n=5; age: 60, 66, 70, 76, 86), GALNT14 expression is basally located near the nucleus, in both flat and columnar glandular epithelium. But if present, the expression in those individual cells is generally weak (**Figures 3A, B**). Glandular epithelium, which looks multilayered due to sectioning, appears more GALNT14-positive in some cases. In addition, GALNT14 is expressed in skeletal muscle in areas distant from glands (**Figure 3D**). Furthermore, the stroma does not show any specific staining (**Figure 3C**), whereas a staining of the endothelium of small blood vessels and perikarya of neurons can be detected, as already shown in healthy tissue (**Figure S3**). In general, the expression pattern resembles the one in healthy prostate tissue at the corresponding age.

Besides, one rare sample of an embryonic rhabdomyosarcoma (ERMS) was available for this study. After determining the tumor cells by desmin, marking intact glandular tissue with cytokeratin 7 (CK7) and excluding macrophages by CD68 (**Figure S4**), the distribution and localization of GALNT14 was investigated (**Figure 3**). Adjacent normal tissue shows intact glandular tissue with a typical weak GALNT14 signal, whereas tumor tissue shows a high number of GALNT14-positive cells with large nuclei.

### 2.4 GALNT14 expression in prostate carcinoma

**Figure 4:**
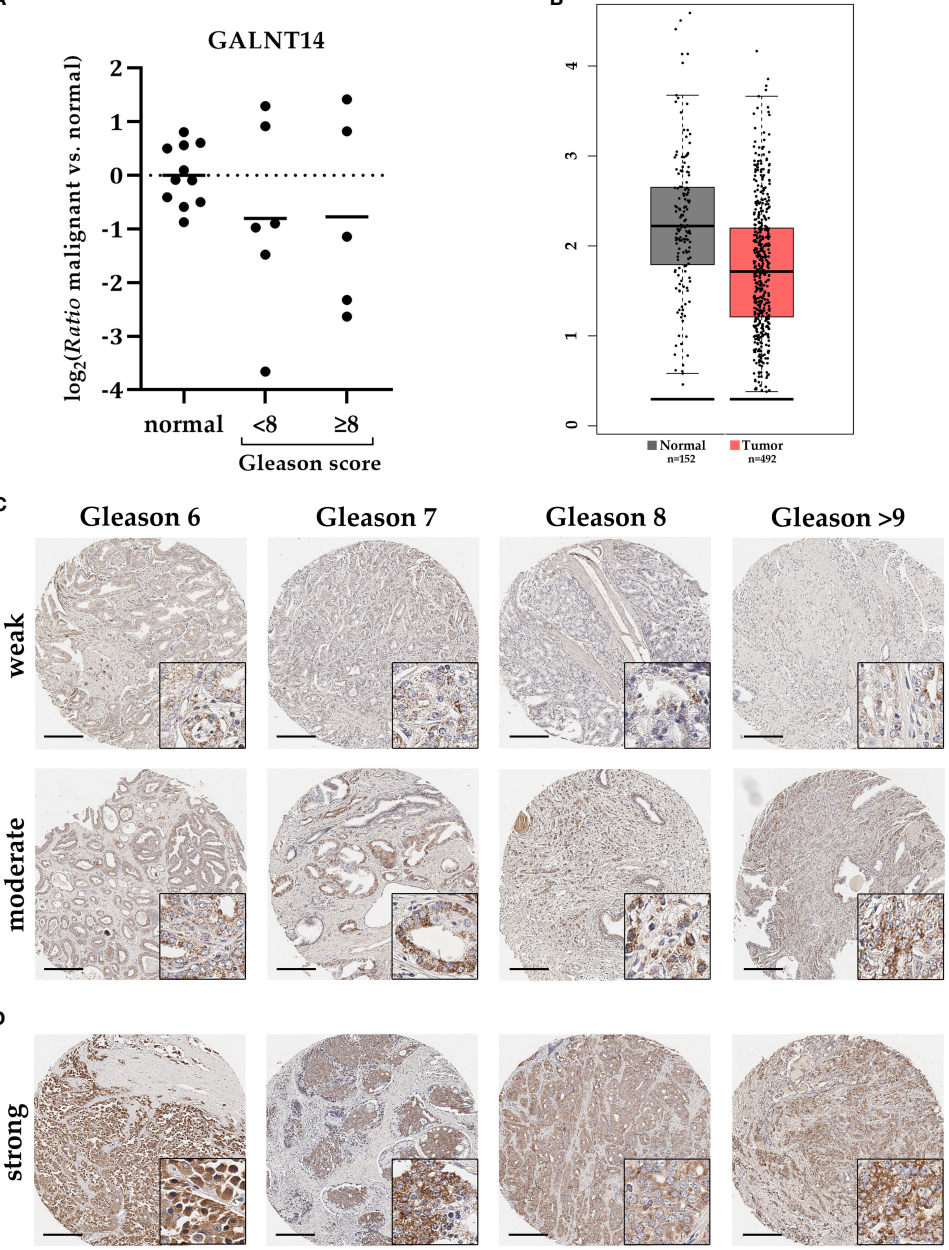
GALNT14 expression in PCa samples with different Gleason score. **(A)** The relative expression change (ratio) of GALNT14 in cryopreserved PCa samples with Gleason score less than (n=6) or starting from 8 (n=5) compared to adjacent normal tissue (n=11) were determined by qRT-PCR. Expression in normal tissue is shown as distribution around the mean. GAPDH and HPRT1 both served as reference genes. GALNT14 expression is repressed on average in PCa regardless of the Gleason score. **(B)** This repression is also supported by the GEPIA Database (adjusted graph). **(C)** GALNT14 expression was assessed using tumor microarrays with 203 PCa specimens with different Gleason scores. Two representative samples per Gleason score are shown. GALNT14 is detected particularly in tumor cells with mostly weak (upper row) or moderate (lower row) intensity. Here, no direct correlation with Gleason score could be detected. **(D)** Few samples (n=9) show an intense GALNT14 expression. Gleason score ranged from 8 to 9. Staining is concentrated on tumor cells and is localized near the nucleus. Scale bar, 200 µm.

To characterize the role of GALNT14 in PCa, first the general expression of GALNT14 on RNA level was determined using qRT-PCR. Six cryoconserved PCa tissue samples with Gleason score below 8 and five with 8 or above were available. For quantification, GALNT14 expression was detected in adjacent normal tissue from the same specimen (**Figure 4A**). Although GALNT14 expression in individual samples varies significantly between induction and repression, it is repressed by about 40% on average, independent of the Gleason score (log_2_ ≈ -0.8). A comparable result is recorded by the database GEPIA (**Figure 4B**).

To compare these results with the expression in PCa tissue, four tissue microarrays (TMAs) were immunohistochemically stained with a total of 203 PCa punch biopsies with different Gleason scores from 180 individual patients. **Table 4** summarizes the number of all samples analyzed by Gleason score. There were two punch biopsies per patient, both of which were included in the analysis if they differed in Gleason score (23 patients).

**Table 4:** Overview of evaluated samples for GALNT14 expression and number of patients depending on Gleason score.

Gleason score	Sample size	Number of patients
**3 + 3 (6)**	39	32
**3 + 4 und 4 + 3 (7)**	55	52
**4 + 4 (8)**	48	45
**≥ 4 + 5 (≥9)**	61	51

GALNT14 staining has primarily a spotted appearance near the nuclei of tumor cells (**Figure 4C**). Consistent with the previously described qRT-PCR data, GALNT14 expression in PCa tissue varies. Overall, a ratio of approximately two-thirds with weak staining and one-third with moderate staining per Gleason score emerged (**Table 5**). In addition, nine punch biopsies were detected that showed strong GALNT14 expression. Four representative samples are shown in **Figure 4D**.

**Table 5:** Overview of the expression intensity (weak, moderate, strong) of GALNT14 in tumor cells dependent on the Gleason score.

	Expression intensity (sample size and percentage)		
Gleason score	weak	moderate	strong
**3 + 3 (6)**	29 (74%)	10 (26%)	/
**3 + 4 und 4 + 3 (7)**	34 (62%)	18 (33%)	3 (5%)
**4 + 4 (8)**	31 (65%)	15 (31%)	2 (4%)
**≥ 4 + 5 (≥9)**	43 (70%)	14 (23%)	4 (7%)

In summary, GALNT14 is weakly to rarely moderately expressed and in very few exceptions even strongly expressed in PCa regardless of the Gleason score.

## 3 Discussion

This study aimed to investigate the stromal-epithelial interaction in PCa via soluble factors. For this purpose, a co-culture system was established in which the prostate carcinoma cell line LNCaP and the primary stromal cells p21 only communicated via soluble factors within the culture medium. Three individually defined time points (one day, three days, and seven days) were chosen for gene expression analysis as it is not exactly known when a change in mRNA expression profiles would manifest due to paracrine action. Finally, RNA sequencing revealed several significantly altered genes (p ≤ 0.05) with at least a two-fold induction or repression. LNCaP cells were used for the primary analysis of stromal-tumor interaction because they still represent the most typical type of prostate carcinoma due to their expression of the androgen receptor (AR) and their hormone dependence unlike other cell lines. For example, 22Rv1 cells are also AR-positive, but they express a constitutively active form and can therefore barely be stimulated with androgens. DU145 cells are AR-negative, i.e. castration-resistant. PC-3 and NCI- H660 cells also belong to castration-resistant cell lines and additionally express neuroendocrine markers.

Regarding the expression change independent of the duration, no significantly deregulated genes were detected for LNCaP cells, and only five induced genes for p21 cells. For instance, ANKRD37 and MIR210HG are considered as target gene of the transcription factor HIF-1, which itself is induced during hypoxia (20, 21). NDRG1, an α/β-hydrolase, is described to have a tumor suppressive character in PCa and to be involved in the regulation of androgen receptor signaling (22, 23). ERRFI1 is a negative regulator of the growth factor EGFR and has been identified in breast carcinoma as a gene altered by hypoxia (24). Also, the channel protein AQP1, which ensures passive transport of water, is induced upon hypoxia in prostate carcinoma cells (25). In conclusion, p21 cells exhibit a hypoxia-like gene expression pattern during co-cultivation with LNCaP cells. However, a corresponding induction of *HIF* genes in p21 cells after co-cultivation could not be detected by RNA sequencing which argues for an alternative regulation of gene expression.

Next, changes in gene expression at specific time points, i.e. day 1, 3 or 7, should be considered separately. For this purpose, genes were compared between the control of one day and the corresponding co-culture. Again, only a differential expression profile was observed in p21 cells and not in LNCaP cells. Gene expression changes were detected after three days at first (11 genes) and the number of deregulated genes increased markedly after seven days (98 genes). The latter were analyzed using the DAVID and STRING bioinformatics databases to map those genes to specific metabolic pathways. At least eight of 98 genes were associated with the “HIF-1 signal transduction pathway” (KEGG, hsa04066, EASE score ≤0.05). This could be a results of poor cultivation conditions due to excessive cell density with excessive nutrient consumption. Replenishment or replacement of culture medium was not included to avoid additional cellular stress and to avoid deprivation of critical soluble factors. On the contrary, hypoxia is considered a typical condition in the tumor microenvironment and would argue for interaction between the benign p21 cells and malignant LNCaP cells (26, 27). As only a fraction of analyzed genes are represented, the importance of this signal transduction pathway should still be critically viewed.

The last comparison aimed to reveal whether gene expression changes occur over time. Therefore, gene expression on day 1 was compared with that on day 7 for both control and co-culture. In addition, matching genes were excluded since they were defined as not specific for co-cultivation. This resulted in numerous co-culture-specific deregulated genes for both p21 and LNCaP cells. Validation of several genes by qRT-PCR in three independent co-culture experiments further confirmed the reproducibility of the experiment and deregulation of gene expression. Moreover, this was independent on the spatial orientation of co-cultured cells which argues for an apical as well as basolateral secretion of soluble factors. Analysis of genes altered in p21 cells via DAVID highlighted the biological process “extracellular matrix organization” (GO:0030198, EASE score ≤0.05). A total of 27 out of 890 assigned genes were subordinated to this process and collagen genes seemed to played an important role (**Table S2**). COL4A4 was one of the highest induced genes and represents an important structural component of the basal lamina. Generally, an enhanced production of collagen could correlate with tissue stiffness and thus with cancer progression (28, 29). Although *RNF215* showed the highest induction in LNCaP cells, it was not chosen as candidate gene because of the general lack on study data. RNF215 expression is only reported to be upregulated by viral infection in human macrophages and might play an important role in the pathogenesis of autoimmune diseases (30). In addition, RNF215 is introduced within a four−gene methylation signature to predict the survival outcome of head and neck squamous cell carcinoma (31). Instead of *RNF215*, *GALNT14* was further analyzed as one of the highest induced gene after co-cultivation. The N-acetylgalactosaminyltransferase GALNT14 mainly initiates and regulates the Mucin-type O-glycosylation. The mucins MUC2, MUC5AC, MUC7 as well as MUC13 are known target proteins of GALNT14 (15). Regarding this, the RNA sequencing results just gave an indication of the involvement of MUC3A in stromal-epithelial interaction. However, GALNT14 only leads to posttranslational O-glycosylation of mucins and not to their increased expression. Beyond, GALNT14 plays a role in migration, invasion, and proliferation of cells particularly in the development of breast carcinoma and supports epithelial-mesenchymal transition and metastasis (32–34). Furthermore, increased GALNT14 expression is associated with resistance to therapeutics in breast carcinoma (35, 36). Regarding PCa, an *in vitro* study demonstrates that GALNT14 was upregulated in the castration-resistant prostate carcinoma cell line PC-3, which stably expressed the metastasis suppressor CD82 (37).

In this study, prostate tissue of different origin was immunohistochemically stained to analyze the localization and distribution of GALNT14-positive cells. During prostate development, GALNT14 showed only very weak expression in epithelial and stromal cells. Although the organogenesis is probably not directly dependent on GALNT14, Shamseldin and colleagues demonstrated the relevance of GALNT14 for general embryogenesis (38). A high lethality in offspring of consanguineous parents due to a homozygous truncated mutation of GALNT14 could be shown. Besides, the impact of the GALNT gene family for the viability of Drosophila melanogaster was already evidenced in 2002 (39). Furthermore, an age-dependent reduction of GALNT14 expression in epithelial and stromal cells of normal and BPH tissue was observed. A major change during aging of an adult male is the hormonal transition as the ratio of testosterone and estrogen shifts toward estrogen (40). Since hormones have a major impact on cellular properties, it is reasonable to assume that GALNT14 expression also is hormone dependent. In line with this, one study showed androgen-regulated glycosylation by GALNT7 as an important modification for the viability of prostate carcinoma cells (41). Regarding the GALNT14 expression in skeletal muscle, no specific studies are known, yet. Only the altered posttranslational O-glycosylation of α-dystroglycan has been linked to the development of various hereditary muscular dystrophies (42, 43). This knowledge might also explain the presence of GALNT14 in tumor cells of embryonal rhabdomyosarcoma of the prostate as this myogenic neoplasm originates from muscle cells. Due to the rarity of this disease, specimens and research results are very limited. To sum up, the described results give a tendency of age dependent GALNT14 expression in human prostate tissue of different developmental stages as well as pathology. Due to the limited number of samples and the lack of a broad coverage of different age groups, it can only be assumed that this reduction results as a cause of a postpubertal effect.

Furthermore, general GALNT14 expression in PCa tissue compared with adjacent normal tissue was examined at RNA level. On average, GALNT14 was downregulated and showed no dependence on the Gleason score. Results from the GEPIA database as well as the subsequent immunohistochemical analysis confirmed this trend. Expression in tumor tissue ranged from weak to moderate, with GALNT14 predominantly localized in perinuclear granular structures. This further verified the predominant expression of GALNT14 in the Golgi apparatus. In addition, strongly upregulated GALNT14 expression was detected for nine punch biopsies. How these samples differ from the others cannot be explained with the present data. Additional known clinical parameters, such as increase of PSA concentration, occurrence of relapses or metastases as well as the use of anti-androgen therapy, do not allow to assign the samples to a specific group. However, a recent study shows that GALNT14 is involved in the regulation of apoptosis and ferroptosis in ovarian cancer and contributes to the development of chemoresistance (44). Chemoresistant ovarian cancer cells exhibited increased GALNT14 expression, which led to enhanced tumor cell viability via the EGFR/mTOR signaling pathway. Overexpression of GALNT14 due to decreased DNA methylation is also associated with poor prognosis for lung adenocarcinoma patients (45).

Future analyses for a deeper understanding of stromal-epithelial interaction in the prostate via soluble factors should include protein analysis of cell culture supernatant from co-culture experiments. This data would complement the RNA sequencing data and possibly link the individual gene expression changes in the different cell lines to uncover specific signal transduction pathways. In addition, this knowledge may help to understand the function of GALNT14 and to elucidate GALNT14-initiated signaling pathways involved in prostate cancer as an increased GALNT14 expression is clearly associated with adverse effects across tumor entities.

## 4 Materials and methods

### 4.1 Cell lines and co-culture system

LNCaP cells (*Lymph Node Carcinoma of Prostate* cells; RRID : CVCL_0395; Sigma-Aldrich, Hamburg, Germany) were grown in RPMI 1640 medium (*Roswell Park Memorial Institute* 1640; Thermo Fisher Scientific, Oberhausen, Germany) supplemented with 10% heat-inactivated FCS (Life Technologies (Gibco), Thermo Fisher Scientific, Oberhausen, Germany), 100 U/ml penicillin, 100 μg/ml streptomycin and 1 mM pyruvate (Thermo Fisher Scientific, Schwerte, Germany). LNCaP cells were last authenticated in 2020 by ATCC performing STR Profiling following ISO 9001:2008 and ISO/IEC 17025:2005 quality standards.

Primary p21 cells (46) were cultivated in the same medium used for LNCaP cells but additionally supplemented with 2.5% HEPES. In general, cells were grown at 37°C in 5% CO_2_ atmosphere. To test cell lines for mycoplasma infection, they were fixed on a coverslip with methanol for 15 min, mounted on a slide with a DAPI-containing Mounting Medium (VECTASHIELD^®^, Vector Laboratories, Burlingame, USA) and examined with a fluorescence microscope (Nikon Eclipse Ni, Amsterdam, Netherlands).

In order to investigate the indirect stromal-epithelial interaction via soluble factors, a co-cultivation system using Millicell hanging cell culture inserts (Merck, Darmstadt, Germany) with a permeable PET membrane and a pore size of 1.0 µm was established for 6-well plates. According to the duration of cultivation, different amounts of cells were seeded into the culture plate or the hanging insert (**Table S3**). After 24 h, the insert including the cells was transferred onto the cell containing well to build up the co-culture system. Cells were co-cultivated for one, three and seven days without media exchange before total RNA was isolated. As controls, the same cell line was co-cultivated with itself (**Figure 1A**).

### 4.2 Human prostate specimens and tissue microarrays

Prostate tissue was acquired through various collaborations. Fetal tissue (n=2), normal tissue of different age (n=9), BPH (n=5) and rhabdomyosarcoma (n=1) were supplied by the Institute of General and Special Pathology at Saarland University Hospital in Homburg. The approval of the ethics committee of the University of Duisburg-Essen has been obtained (Ref: 18-7959-BO). A total of six samples of fetal prostate tissue were obtained from the research group of Dr. Laurence Baskin at the Department of Urology, University of California, San Francisco (UCSF). The use of the tissue has been approved by the local ethics committee (also included in Ethics Application 18-7959-BO). Prostate cancer tissue was obtained through the following collaborations: Tissue biobank of the Comprehensive Cancer Center (CCC-ER EMN) of the University Hospital Erlangen in cooperation with Prof. Dr. Helge Taubert and Dr. Sven Wach (project no. 2019-112; n=11 cryoconserved); Department of Urology at the Community Hospital Karlsruhe or from patients recruited from the EMPaCT tumor bank (European Multicenter Prostate Cancer Clinical and Translational Research Group) in cooperation with Prof. Dr. Martin Spahn, PD Dr. phil. Marianna Kruithof-de Julio and Dr. phil. Eugenio Zoni (n=203 of N=180 paraffin-embedded) (47). Tissue microarrays (TMAs) were generated by multiple tumor samples derived from the index lesion including more differentiated areas of each tumor as well as matched lymph node metastasis from previously untreated patients. TMAs were also characterized with several tumor relevant genes (e.g. AR, PTEN, p53, MLH1, CD44, ALDH1, chromogranin A, and synaptophysin) and the TMPRSS2-ERG gene fusion (48, 49). Approval was obtained from the ethics committee in Bern (reference: KEK Bern No. 128/2015).

Pathological evaluation of all prostate tissue samples was performed by pathologists at the respective sites.

### 4.3 RNA isolation

Total RNA was extracted from cell lines and cryoconserved tissue sections using the miRNeasy Mini Kit (Qiagen, Hilden, Germany) according to the manufacturer’s instruction. RNA concentration and quality was controlled on the BioPhotometer (Eppendorf, Hamburg, Germany). Furthermore, the RNA was transcribed into cDNA to perform PCR for GAPDH detection. A successful amplification confirmed the integrity of the RNA molecules. In addition, to exclude DNA contamination, PCR was performed with RNA, instead of the corresponding cDNA, for each sample (**Figure S1A**).

### 4.4 RNA sequencing

Sample preparation using the QuantSeq 3’ mRNA-Seq Library Prep Kit from Lexogen (Vienna, Austria) and RNA sequencing via the Nextseq™ 500 (Illumina, Berlin, DE) were performed by the Genomics & Transcriptomics Facility (GTF) of the University Hospital Essen. Quality controlled samples (n=4) were finally sequenced using the Lexogen technology according to the manufacturer’s specifications. Unlike conventional RNA sequencing, this sequencing is dependent on the poly(A) end of the mRNA. The primary processing of the sequencing data and the comparative analyses between control and co-culture were also performed by GTF. The BlueBee analysis platform (Bluebee Holding B.V., Rijswijk, Netherlands) was used for this purpose.

### 4.5 cDNA synthesis and quantitative real-time PCR

mRNAs were transcribed into complementary DNA (cDNA) using the High Capacity cDNA Reverse Transcription Kit (Applied Biosystems, Darmstadt, Germany). 1 µg isolated RNA was used and cDNA synthesis was proceeded according to the manufacturer’s protocol. Prior, possible DNA contaminations were removed by incubating the RNA with 1 U/µl DNase I (Invitrogen, Darmstadt, Germany).

Next, the relative gene expression was analyzed using the my-Budget 5X Evagreen^®^ QPCR-Mix II (Biobudget, Krefeld, Germany) according to manufacturer’s instruction and the real-time PCR detection system qTower^3^G (Analytic Jena, Jena, Germany). For relative quantification, the expression of the target genes was normalized to the expression of the reference genes GAPDH and HPRT1 using the ΔΔCt method. For each oligonucleotide pair, an additional control without template was analyzed (Non Template Control, NTC) and all preparations were carried out in duplicates. The thermal cycling conditions were as follows: 95°C for 15 min followed by 38 cycles of 95°C for 15 sec, 60°C for 30 sec and 72°C for 30 sec. The melting curve analysis ensured the purity and specificity of PCR. **Table S4** lists the respective oligonucleotide sequences.

### 4.6 Immunohistochemical and immunofluorescence staining

5 µm sections of formalin-fixed and paraffin embedded prostate tissue were used for immunostaining. For antigen unmasking, deparaffinized and rehydrated slices were incubated for 30 min in citrate buffer at 96°C. Permeabilization was performed with 0,1% Triton X-114 for 10 min. After blocking with 1% BSA, primary antibodies were diluted in 0,5% BSA/PBS and incubated over night: Rabbit anti-GALNT14 (1:150, BS- 11018R, Bioss Antibodies, Woburn, USA), mouse anti-Cytokeratin 7 (1:50, M7018, Dako, Jena, Germany), mouse anti-Desmin (1:100, M0760, Dako, Jena, Germany), mouse anti-CD68 (1:100, SAB5500070, Sigma-Aldrich, Hamburg, Germany), mouse anti-CHGA (1:100, ab715, Abcam, Cambridge, Great Britain).

On the following day, biotin-conjugated secondary antibodies were incubated for 60 min at room temperature (1:250 in 0.5% BSA/PBS): Biotinylated swine anti-rabbit (E0353, Dako, Jena, Germany), biotinylated rabbit anti-mouse (E0354, Dako, Jena, Germany).

For immunofluorescence staining, tissue sections were additionally treated with MaxBlock™ Autofluorescence Reducing Reagent Kit (Biozol, Eching, Germany) according to the manufacturer’s instructions. A Cy™3-conjugated streptavidin (1:200; Jackson Immuno Research, Pennsylvania, US) was incubated to visualize the antigen and DAPI (Jackson Immuno Research, Pennsylvania, US) to stain the DNA (both 1:200 in 0.5% BSA/PBS, 30 min at room temperature). In the case of a double incubation, binding sites within the tissue were blocked again with 1% BSA for 60 min. The secondary antibody (mouse anti-CD68, 1:100, SAB5500070, Sigma-Aldrich, Hamburg, Germany) was then visualized using the Alexa Fluor^®^ 488 AffiniPure Goat Anti-Mouse IgG (H+L) (Jackson Immuno Research, Pennsylvania, US). Fluoromount-G (Southern Biotech/Biozol, Eching, Germany) was finally used to cover the sections. Detection was performed using the Eclipse Ni microscope (Nikon, Amsterdam, Netherlands) and the associated NIS Elements AR software.

For immunohistochemistry, tissue sections were treated with a complex of streptavidin and biotinylated HRP (VectaStain^®^ Elite^®^ ABC Kit, Peroxidase Kit, Vector Laboratoriers; 1:250 in 0.5% BSA/PBS) for signal enhancement (30 min at room temperature). Finally, antigens were visualized by adding diaminobenzidine (DAB; Sigma Aldrich, Hamburg, Germany) as HRP substrate. Hematoxylin-stained cell nuclei and HCl was used for differentiation. After dehydration, tissue sections were mounted with Xylene Substitute Mountant (Thermo Fisher Scientific), finally scanned at a magnification of 400x using the Aperio ScanScope Slide Scanner (Leica, Wetzlar, Germany) and analyzed via the corresponding ImageScope software.

For each staining, a positive tissue control and a negative isotype control (IgG fraction of non-immunized rabbits or mouse anti-rat-CEACAM1 (IgG κ)) were carried along with the tissue to be examined (**Figure S2**).

### 4.7 Gene ontology and pathway enrichment analysis

For functional annotation clustering and pathway analysis of deregulated genes after stromal-epithelial interaction on day 7 in p21 cells, the Database for Annotation, Visualization and Integrated Discovery (DAVID, https://david.ncifcrf.gov) was used (50, 51). This tool refers to several public genomic resources like NCBI, Uniprot, Ensembl, KEGG or Reactome. We analyzed the gene lists with default parameters, except an EASE score of 0.05. Additionally, the database STRING (https://string-db.org) was used for the same purpose with default adjustments (52).

### 4.8 Visualization and statistical analyses

Data of qRT-PCR were analyzed and visualized using SigmaPlot 13 (Systat, Erkrath, Germany). Results are represented as mean ± standard deviation from three independent measurements. GraphPad Prism 9 (GraphPad Software, San Diego, California USA) was used to visualize induced and repressed genes in cell lines in a Volcano Plot. The database GEPIA (Gene Expression Profiling Interactive Analysis) was used to perform prostate cancer/normal differential expression analysis using default adjustments (53).

## 5 Conclusions

In conclusion, this study provides a new basis for a better understanding of the complex communication between stromal and epithelial cells in the prostate and prostate carcinoma, respectively. Illuminating the paracrine interaction created a new perspective and revealed metabolic pathways that may have a relevance in the development and progression of prostate cancer. For example, components of the HIF-1 pathway were upregulated in stromal cells which might further underline the relevance of hypoxia during tumorigenesis as already described for prostate carcinoma (54). Moreover, for prostate carcinoma an induced expression of the N-acetylgalactosaminyltransferase GALNT14 is shown in rare cases of pathology which could help to further characterize this heterogenous tumor. Overall, this work shows the importance of regarding cell-cell communication to learn about prostate cancer development. Accordingly, the tumor microenvironment must be considered in addition to the study of the tumor cells themselves.

## Data availability statement

The original contributions presented in the study are included in the article/**Supplementary Material**. Further inquiries can be directed to the corresponding author. Sequencing data is publicly available on Gene Expression Omnibus (GEO) with GEO accession GSE210988.

## Ethics statement

The study was conducted according to the guidelines of the Declaration of Helsinki, and approved by the Kantonale Ethikkomission (KEK) Bern (128/2015) and the ethics committee of the University of Duisburg-Essen (Ref: 18-7959-BO). The patients/participants provided their written informed consent to participate in this study.

## Author contributions

Conceptualization, EC. Methodology, EC. Validation, EC and MW. Formal analysis, EC. Investigation, EC and MW. Resources, MWa, MS, MK and SW. Data curation, EC. Writing—original draft preparation, EC. Writing—review and editing, JD, MW, GW, SW. Visualization, EC. Supervision, EC, JD and GW. Project administration, EC, JD and GW. Funding acquisition, GW. All authors contributed to the article and submitted and approved the submitted section.
