# Supplementary material for: Stromal-epithelial interaction induces GALNT14 in prostate carcinoma cells
Source: Front Oncol. 2023 Aug 21;13:1212585. doi: 10.3389/fonc.2023.1212585 (PMC10475991; doi:10.3389/fonc.2023.1212585)
Supplement: Supplementary file 1 [file DataSheet_1.docx]

Supplementary Material

Stromal-epithelial interaction induces *GALNT14* in prostate carcinoma cells

Elena D. Czyrnik^1^, Marc Wiesehöfer^1^, Jaroslaw T. Dankert^1^, Sven Wach^2^, Mathias Wagner^3^, Martin Spahn^4,5^, Marianna Kruithof de Julio^6,7^, and Gunther Wennemuth^1,^*

*** Correspondence:** Gunther Wennemuth: gunther.wennemuth@uk-essen.de

# Supplementary Data

## Supplementary Figures

**
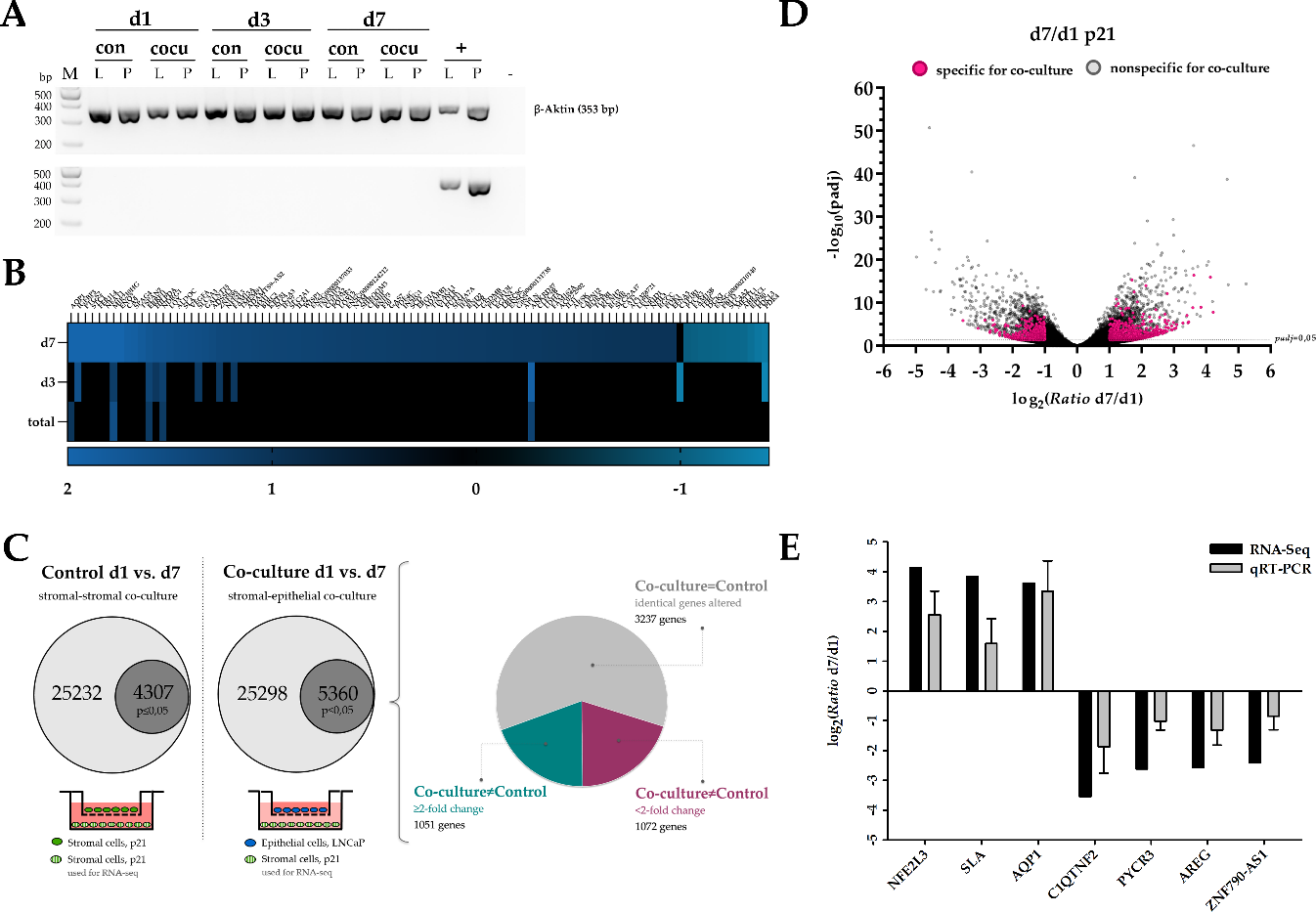
**

**Figure S1.** (**A**) RNA used for RNA sequencing was first quality-controlled by reverse transcriptase PCR (RT-PCR) (n=4). Shown is a representative agarose gel for β-actin detection in co-cultured LNCaP and p21 cells (upper gel). To exclude DNA contamination, RT-PCR with DNase-digested RNA, instead of cDNA, was performed for the same samples. Absence of specific bands confirmed the purity of the RNA samples (lower gel). M, marker; L, LNCaP; P, p21; con, control; cocu, co-culture; +, positive control. (**B**) Visualization of differentially expressed genes in p21 cells after co-cultivation with LNCaP cells on day 3 and day 7 as binary logarithm (p≤0.05, at least two-fold change). On day 1, there was no significant gene expression change. Furthermore, the comparison between all controls and co-cultures regardless of the duration of co-cultivation is shown (all). Except for AQP1, gene expression was equally altered after three and seven days. Dark blue, induced genes; light blue, repressed genes; black, no expression change present. (**C**) Schematic illustration of defining deregulated co-culture-specific genes in p21 cells after seven days of co-cultivation. Significantly deregulated genes for control and co-culture are highlighted (p≤0.05, dark grey). The pie chart additionally illustrates the distribution of identical non co-culture-specific genes (gray, 3237 genes) as well as genes with less than a two-fold expression change (magenta, 1072 genes) and at least two-fold induction or repression (cyan, 1051 genes). (**D**) Volcano-Plot visualizes all 25298 deregulated genes in p21 cells after co-cultivation for the comparison day 1 and day 7. Relative expression change (ratio) is plotted as binary logarithm and the corresponding significance (adjusted p-value/ padj) as negative decadic logarithm. The dashed line marks the limit of biological significance of p≤0.05 (‑log10>1.3). The 1051 induced and repressed co-culture specific genes with at least two-fold change (from log2>1 and log2<-1, respectively) are highlighted (magenta). d1, day 1; d7, day 7 (**E**) Validation of RNA sequencing data by quantitative real-time PCR (qRT PCR) of three independent co-culture experiments. Binary logarithm with standard deviation is indicated.

**
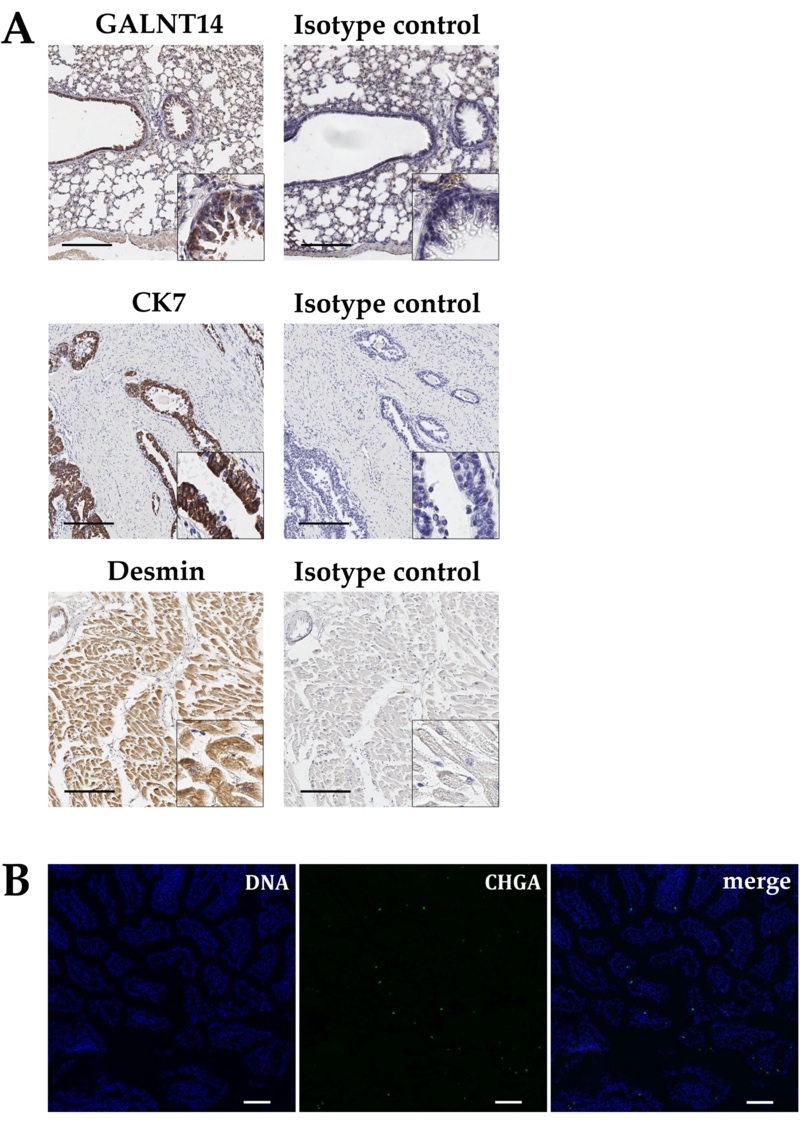
**

**Figure S2.** Control immunhistochemistry to confirm the reliability of stainings. (**A**) For rabbit-anti-GALNT14 antibody murine lung tissue, for mouse-anti-Cytokeratin 7 antibody human prostate tissue and for mouse-anti-Desmin antibody human heart tissue was used. FFPE control tissue was processed as the tissue of interest. For negative or isotype controls, IgG fraction of non-immunized rabbits or mouse anti-rat-CEACAM1 (IgG κ) were used. Scale bar, 200 μm. (**B**) Control immunofluorescence staining was performed with mouse-anti-CHGA antibody using human small intestine tissue. DAPI was used to visualize the DNA. Scale bar, 100 µm.

**
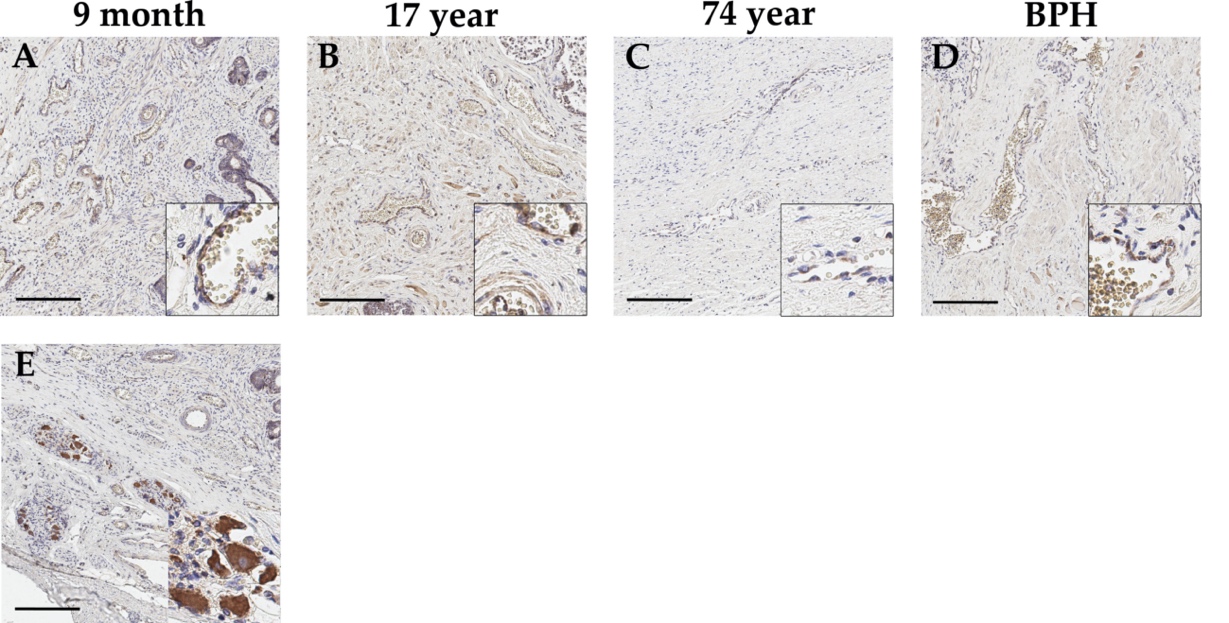
**

**Figure S3.** Representative immunohistochemical staining with Anti-GALNT14 antibody of healthy prostate tissue differing in age and BPH tissue. (**A-D**) The endothelium of small vessels appeared to be moderately GALNT14-positive (**E**) while perikarya of neurons showed a very strong staining. Scale bar, 200 μm.


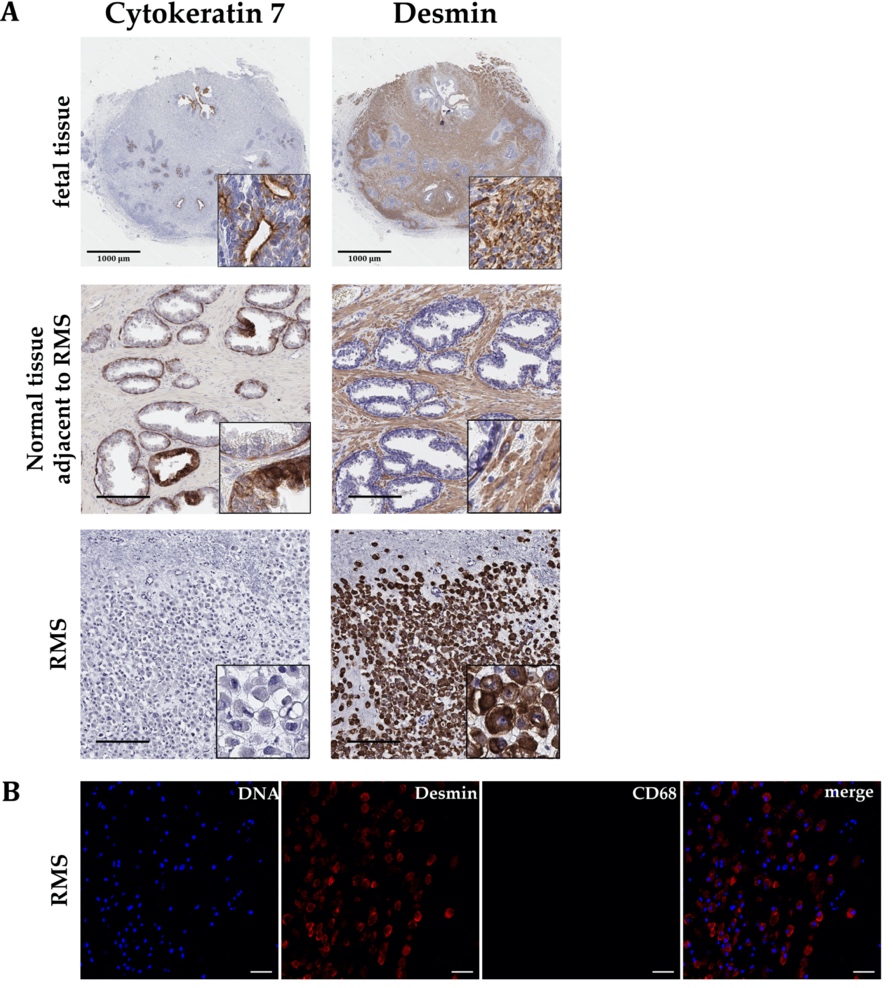


**Figure S4.** Representative immunohistochemical staining of fetal prostate tissue (upper row) and rhabdomyosarcoma (RMS) with adjacent normal tissue. (**A**) Staining with anti-cytokeratin 7 antibody was used to identify the glandular epithelium. Desmin expression additionally visualized muscle cells in the stroma. Scale bar, 200 μm. (**B**) Double immunofluorescence staining was performed to detect tumor cells by Desmin staining and potential macrophages by CD68 staining in RMS. The absence of the macrophage marker CD68 confirmed the exclusive presence of desmin-positive tumor cells. Scale bar, 50 μm.

## Supplementary Tables

**Table S1.** The 20 most induced and repressed genes in p21 cells after co-culture with LNCaP cells depending on the duration (comparison of gene expression on day 7 to day 1).

| **Ensembl-No.** | **Gene** |  | **Ratio**^1^ | ***p*-value** |
| --- | --- | --- | --- | --- |
| ENSG00000174697 | Leptin | *LEP* | 18,63 | 1,75*10^-8^ |
| ENSG00000050344 | **Nuclear Factor, Erythroid 2 Like 3** | ***NFE2L3*** | 17,54 | 1,16*10^-16^ |
| ENSG00000155926 | **Src Like Adaptor** | ***SLA*** | 14,28 | 1,20*10^-9^ |
| ENSG00000240583 | **Aquaporin 1** | ***AQP1*** | 12,26 | 4,26*10^-17^ |
| ENSG00000081052 | Collagen type IV Alpha 4 chain | *COL4A4* | 11,99 | 1,53*10^-9^ |
| ENSG00000129521 | Egl-9 family hypoxia inducible factor 3 | *EGLN3* | 10,83 | 4,96*10^-6^ |
| ENSG00000107159 | Carbonic Anhydrase 9 | *CA9* | 10,59 | 9,56*10^-8^ |
| ENSG00000235997 | lincRNA 1936 | *LINC01936* | 8,34 | 7,26*10^-5^ |
| ENSG00000158445 | Potassium voltage-gated channel subfamily B member 1 | *KCNB1* | 8,10 | 1,11*10^-4^ |
| ENSG00000255418 | lincRNA 2718 | *AC090092.1* | 7,91 | 4,16*10^-6^ |
| ENSG00000145861 | **C1q and TNF related 2** | ***C1QTNF2*** | 0,09 | 1,47*10^-6^ |
| ENSG00000257122 | RRN3 Pseudogene 3 | *RRN3P3* | 0,16 | 1,85*10^-5^ |
| ENSG00000104524 | **Pyrroline-5-Carboxylate Reductase 3** | ***PYCR3*** | 0,16 | 1,03*10^-6^ |
| ENSG00000109321 | **Amphiregulin** | ***AREG*** | 0,17 | 1,17*10^-5^ |
| ENSG00000279821 | TEC | *AC145098.2* | 0,17 | 1,43*10^-4^ |
| ENSG00000225972 | MT-ND1 Pseudogene 23 | *MTND1P23* | 0,17 | 5,42*10^-4^ |
| ENSG00000125319 | Homologous Recombination factor with OB-fold (HROB) | *C17orf53* | 0,18 | 2,25*10^-4^ |
| ENSG00000267254 | **ZNF790 antisense RNA 1** | ***ZNF790-AS1*** | 0,19 | 1,80*10^-3^ |
| ENSG00000143942 | ChaC glutathione specific gamma-glutamylcyclotransferase 2 | *CHAC2* | 0,21 | 3,54*10^-3^ |
| ENSG00000227053 | MUC12 antisense RNA 1 | *AC105446.1* | 0,21 | 2,76*10^-5^ |

^1^ Relative expression change (day 1 vs. day 7 of co-culture). Highlighted genes were validated by real-time PCR.

**Table S2.** List of deregulated genes in p21 cells after co-cultivation with LNCaP cells depending on the duration (comparison of gene expression on day 7 to day 1) attributed to the biological process "Extracelullar matrix organisation" (GO:0030198).

| **Ensembl-No.** | **Gene** |  | **Ratio**^1^ | ***p*-value** |
| --- | --- | --- | --- | --- |
| ENSG00000081052 | Collagen Type IV Alpha 4 Chain | *COL4A4* | 11.99 | 1.53*10^-9^ |
| ENSG00000171812 | Collagen Type VIII Alpha 2 Chain | *COL8A2* | 5.53 | 3.03*10^-5^ |
| ENSG00000163638 | ADAM Metallopeptidase With Thrombospondin Type 1 Motif 9 | *ADAMTS9* | 4.93 | 2.18*10^-6^ |
| ENSG00000166106 | ADAM Metallopeptidase With Thrombospondin Type 1 Motif 15 | *ADAMTS15* | 4.67 | 4.15*10^-3^ |
| ENSG00000154175 | ABI Family Member 3 Binding Protein | *ABI3BP* | 4.20 | 1.04*10^-2^ |
| ENSG00000060718 | Collagen Type XI Alpha 1 Chain | *COL11A1* | 3.96 | 1.78*10^-2^ |
| ENSG00000130702 | Laminin Subunit Alpha 5 | *LAMA5* | 3.48 | 3.52*10^-4^ |
| ENSG00000116962 | Nidogen 1 | *NID1* | 3.07 | 2.30*10^-5^ |
| ENSG00000163520 | Fibulin 2 | *FBLN2* | 3.05 | 1.44*10^-2^ |
| ENSG00000100767 | Papilin, Proteoglycan Like Sulfated Glycoprotein | *PAPLN* | 2.76 | 1.96*10^-2^ |
| ENSG00000103196 | Cysteine Rich Secretory Protein LCCL Domain Containing 2 | *CRISPLD2* | 2.73 | 3.73*10^-3^ |
| ENSG00000139329 | Lumican | *LUM* | 2.68 | 1.52*10^-4^ |
| ENSG00000168542 | Collagen Type III Alpha 1 Chain | *COL3A1* | 2.68 | 2.12*10^-4^ |
| ENSG00000133110 | Periostin | *POSTN* | 2.57 | 8.52*10^-4^ |
| ENSG00000144810 | Collagen Type VIII Alpha 1 Chain | *COL8A1* | 2.48 | 4.07*10^-2^ |
| ENSG00000134853 | Platelet Derived Growth Factor Receptor Alpha | *PDGFRA* | 2.45 | 3.16*10^-2^ |
| ENSG00000142303 | ADAM Metallopeptidase With Thrombospondin Type 1 Motif 10 | *ADAMTS10* | 2.41 | 2.68*10^-2^ |
| ENSG00000049192 | ADAM Metallopeptidase With Thrombospondin Type 1 Motif 6 | *ADAMTS6* | 2.41 | 1.48*10^-2^ |
| ENSG00000197930 | Endoplasmic Reticulum Oxidoreductase 1 Alpha | *ERO1A* | 2.41 | 1.62*10^-10^ |
| ENSG00000142871 | Cellular Communication Network Factor 1 | *CCN1* | 2.15 | 2.77*10^-5^ |
| ENSG00000130635 | Collagen Type V Alpha 1 Chain | *COL5A1* | 2.14 | 5.36*10^-7^ |
| ENSG00000188153 | Collagen Type IV Alpha 5 Chain | *COL4A5* | 2.05 | 2.50*10^-2^ |
| ENSG00000163359 | Collagen Type VI Alpha 3 Chain | *COL6A3* | 2.05 | 8.87*10^-6^ |
| ENSG00000164761 | TNF Receptor Superfamily Member 11b | *TNFRSF11B* | 2.03 | 3.82*10^-4^ |
| ENSG00000143382 | ADAMTS Like 4 | *ADAMTSL4* | 2.01 | 1.35*10^-2^ |
| ENSG00000141098 | Glucose-Fructose Oxidoreductase Domain Containing 2 | *GFOD2* | 0.47 | 1.17*10^-2^ |
| ENSG00000262406 | Matrix Metallopeptidase 12 | *MMP12* | 0.28 | 1.32*10^-2^ |

^1^ Relative expression change (day 1 vs. day 7 of co-culture).

**Table S3.** Overview of the number of cells to be seeded depending on cell type and cultivation time.

| **Duration of co-culture** | **LNCaP cells** | **p21 cells** |
| --- | --- | --- |
| One day | 7x10^5^ | 1,3x10^5^ |
| Three days | 5x10^5^ | 8x10^4^ |
| Seven days | 2x10^5^ | 6x10^4^ |

**Table S4.** Oligonucleotide pairs and resulting fragment size for quantitative real-time PCR.

| Gene | Sequence (5’-3’), complementary reverse | Fragment size (bp) |
| --- | --- | --- |
| 5‘ AC073611.1 | GAACCTACCTCTGGTGATGG | 94 |
| 3‘AC073611.1 | CCCGAAAAACTCGCTTCCTG |  |
| 5‘AL122023.1 | CATGGGATTCTAAACACCGTATTG | 83 |
| 3‘AL122023.1 | CATACTCAAGTCTGCCTGTCC |  |
| 5‘ AQP1 | CTGGCTGATGGTGTGAACTC | 86 |
| 3‘ AQP1 | GTAGTAGCCAGCACGCATAG |  |
| 5‘ AREG | tgatcctcacagctgttgct | 107 |
| 3‘ AREG | tccattctcttgtcgaagtttct |  |
| 5‘ C1QTNF2 | CGAGGGTAACCACCATGATC | 95 |
| 3‘ C1QTNF2 | GAAGTCCCTGCGAGCAAAG |  |
| 5‘ GALNT14 | TGTCAGTCATCACCTTGTTC | 76 |
| 3‘ GALNT14 | CATTGCTGTCGGTCATCT |  |
| 5‘ GAPDH | TGGAGAAGGCTGGGGCTCAT | 176 |
| 3‘ GAPDH | GACCTTGGCCAGGGGTGCTA |  |
| 5‘ HPRT1 | GCATACCTAATCATTATGCTGAGG | 87 |
| 3‘HPRT1 | CTCGAGCAAGACGTTCAGTC |  |
| 5‘ IZUMO4 | CGCCTGCTTTGGCTATAACT | 102 |
| 3‘ IZUMO4 | ATGCTCGTGTCCTGTTTGTG |  |
| 5‘ NFE2L3 | CTTACAGCCAACTGCACCAG | 75 |
| 3‘ NFE2L3 | CTACTCCTTATCTTCTGTGACTTC |  |
| 5‘ PYCR3 | GACAGGAACCTATGTCACTTTCAA | 94 |
| 3‘ PYCR3 | tggcaaagatgacgagca |  |
| 5‘ RNF215 | TATGGAGGATGGCAGGACTT | 69 |
| 3‘RNF215 | CTGCTGCAGGGGTTTCTG |  |
| 5‘ SLA | TGGCTGGATCGGGTAGGTAA | 144 |
| 3‘ SLA | CCCCATCTTTCCTGGAGCTG |  |
| 5‘ SLC7A11/xCT | TCATGTCCGCAAGCACACTCC | 210 |
| 3‘ SLC7A11/xCT | GGGATGAACAGTGGCACCTTGA |  |
| 5‘ ZNF790-AS1 | ctggatccctcagcagtga | 77 |
| 3‘ ZNF790-AS1 | tctcaggaggagtgggagtg |  |
